# Effects of exercise interventions on memory in depression: a three-level meta-analysis

**DOI:** 10.7717/peerj.20750

**Published:** 2026-02-05

**Authors:** Xiaoling Zhu, Yunong Zhang, Cong Liu, Xing Wang

**Affiliations:** 1 Sports Department, University of Shanghai for Science and Technology, Shanghai, Yangpu, China; 2 Department of Physical Education, Sejong University, Seoul, Republic of South Korea; 3 School of Physical Education, Shanghai University of Sport, Shanghai, Yangpu, China

**Keywords:** Exercise, Memory, Depression, Meta-analysis

## Abstract

**Background:**

Patients with depression have memory impairment. Exercise can improve memory in people with depression. This study employs a three-level meta-analysis to investigate the interventional effects of exercise on verbal and visual memory in patients with depression.

**Methods:**

A systematic electronic search was conducted in China National Knowledge Infrastructure, Wanfang Data, China Biomedicine, PubMed, EMBASE, the Cochrane Library, and Web of Science to identify randomized controlled trials investigating the effects of exercise interventions on memory in individuals with depression, up to July 18, 2024. A three-level meta-analysis based on a random-effects model was performed using R. The risk of bias of the included studies was assessed using the Cochrane Risk of Bias 2 (RoB 2) tool.

**Results:**

A total of 16 studies were included in the analysis. The results indicated a statistically significant but small effect of exercise on verbal memory in patients with depression (*g* = 0.17, 95% confidence interval (CI) [0.02–0.32], *p* = 0.03); however, the 95% prediction interval crossed zero, suggesting that the effect may not be consistent across different settings or future studies; however, the 95% prediction interval crossed zero, suggesting that the effect may not be consistent across different settings or future studies (*g* = 0.27, 95% CI [−0.00–0.54], *p* = 0.05). Exercise intensity significantly moderated the effect of exercise on verbal memory in patients with depression (*F* = 3.39, *p* = 0.04), whereas exercise type, session time, duration, age, and intervention content of the experimental group were not moderating factors (*p* > 0.05). Low-to-moderate intensity (*g* = 0.43, *p* < 0.01), duration ≤12 weeks (*g* = 0.27, *p* < 0.01), and session time ≤60 minutes (*g* = 0.18, *p* = 0.03) of mind-body exercise (*g* = 0.43, *p* < 0.01) were most likely to improve verbal memory in patients with depression. The level of evidence was “moderate”.

**Conclusions:**

Exercise may confer a small improvement in verbal memory among adults with depression, while no clear effect was observed for visual memory. However, further randomized controlled trials are needed to explore the impact of exercise on memory in patients with depression. Research plan was registered in international system evaluation platform PROSPERO (https://www.crd.york.ac.uk/PROSPERO/) (CRD42023473393).

## Introduction

Depression is a mental disorder characterized primarily by a significant low mood. Approximately one in six individuals will experience depression at some point in their lifetime (Gold et al., 2020; Otte et al., 2016). Research indicates that approximately two-thirds of patients with depression exhibit cognitive impairment (Roiser, Elliott & Sahakian, 2012), which is a significant contributor to the onset and persistence of depressive symptoms. This impairment often manifests as memory dysfunction, characterized by deficits in immediate memory, declines in short-term memory, and abnormalities in long-term memory (Rock et al., 2014; Semkovska et al., 2019), adversely affecting patients’ recovery and quality of life. Among these, verbal memory tends to be more consistently and severely affected than visual memory, with meta-analytic evidence indicating larger effect sizes for verbal memory deficits (Lee et al., 2012; Snyder, 2013). This difference suggests that verbal memory may be more sensitive to the effects of depression, highlighting the need to distinguish and separately investigate these memory domains in intervention studies.

To further clarify why these domains may respond differently to interventions, recent neurocognitive research suggests that exercise may exert domain-specific influences on memory (Li et al., 2025; Sun et al., 2025). Exercise-induced neuroplasticity may differentially influence verbal and visual memory *via* distinct neural pathways. Aerobic exercise increases hippocampal brain-derived neurotrophic factor expression, neurogenesis, and dentate gyrus plasticity—changes that preferentially support verbal/episodic memory (Dinoff et al., 2018; Erickson et al., 2011; Han et al., 2023). In contrast, prefrontal-dependent functions such as working memory and visuospatial processing benefit through enhanced cerebral perfusion, synaptic plasticity, and hypothalamic–pituitary–adrenal (HPA)-axis regulation (Stillman et al., 2016). These mechanisms imply that exercise may plausibly exert domain-specific effects on memory subtypes (hippocampal *vs.* prefrontal pathways). This mechanistic rationale aligns with contemporary frameworks emphasizing neuroplasticity, brain-derived neurotrophic factor (BDNF)-mediated hippocampal adaptation, and HPA-axis regulation as core pathways linking exercise with cognitive enhancement (Kandola et al., 2019). A brief conceptual model illustrating these proposed pathways (exercise parameters → neurobiological mediators → verbal/visual memory outcomes) is provided in Fig. S1.

Researchers and the medical communities have recognized exercise as having slight side effects and being easy to implement. Therefore, many guidelines recommend exercise as a means of depression treatment. Exercise can promote the secretion of brain-derived neurotrophic factors in the brain, promote neurogenesis and synaptic plasticity, and improve cognitive functions such as memory (Callaghan, Rouine & O’Mara, 2017; Singh et al., 2025; Sungkarat et al., 2018). Buschert et al. (2019) conducted a study involving aerobic exercise over a duration of 3–4 weeks in patients with depression, demonstrating that aerobic exercise can significantly enhance short-term verbal memory. Similarly, Khatri et al. (2001) implemented a 16-week aerobic exercise program for elderly patients with depression, finding that exercise also positively influenced memory in this population.

Currently, several meta-analyses have explored the impact of exercise on memory in patients with depression. Brondino et al. (2017) employed traditional meta-analysis methods, indicating that exercise can improve verbal memory in patients with depression while showing no effect on visual memory. Conversely, Sun et al. (2018) also utilized traditional meta-analysis, concluding that exercise does not enhance either verbal or visual memory in patients with depression. However, the latest three-level meta-analysis indicates that exercise can improve memory in patients with depression (Ren et al., 2024), yet it does not further investigate the effects on verbal and visual memory. Therefore, it remains contentious regarding the specific impacts of exercise on verbal and visual memory in patients with depression. Previous studies have also suggested that the sources of controversy may be related to the types of exercise included and the failure to differentiate between verbal and visual memory in the overall memory assessment. Additionally, publication bias has been identified (Ren et al., 2024), which may be due to the study’s inclusion of only articles published in English-language journals, excluding other grey literature on verbal memory. Exercise variables such as type, duration, intensity, frequency, and duration of the intervention can influence the effectiveness of exercise in improving cognitive function (Huang et al., 2022; Shimada et al., 2022). Ren et al. (2024) suggested that aerobic exercise at moderate intensity, performed three times per week for less than 60 min and for more than 13 weeks, can improve executive function in patients with depression. Subsequently, they found that moderate-intensity aerobic exercise, conducted three times per week for durations of less than 45 min and between 45–60 min, over 12 weeks or less, can enhance cognitive function (Ren et al., 2023). Conversely, Sun et al. (2018) posited that low-intensity exercise is beneficial for improving cognitive function in patients with depression. Thus, exercise parameters may have a selective impact on the enhancement of cognitive function in patients with depression.

Since this study needs to consider multiple memory indices, multiple effect sizes that meet the inclusion criteria may be reported within the same study. Additionally, given the assumption of independence between effect sizes in traditional meta-analysis, as well as the potential impact of correlations between effect sizes on the combined effect size, this study deliberately forgoes traditional two-level meta-analysis in favor of a more advanced three-level meta-analysis approach (Assink & Wibbelink, 2016), which models variance at the study, outcome, and measurement levels to better handle dependencies among effect sizes. Compared to previous two-level syntheses, this approach improves the accuracy and reliability of pooled estimates, addressing methodological limitations in earlier work. Based on this, the present study expands its scope by including Chinese databases and gray literature alongside English databases. Furthermore, we formulate *a priori* hypotheses that exercise will differentially affect verbal and visual memory in patients with depression, and that exercise parameters such as intensity, duration, and type will moderate these effects. Employing a three-level meta-analysis approach, this study aims to explore the effects of exercise on verbal and visual memory in patients with depression and further investigate the influence of exercise parameters and other factors on memory in this population. The goal is to provide evidence-based guidance for the development of exercise programs aimed at improving memory in patients with depression.

## Methods

This study was conducted by the methods and requirements of the latest Preferred Reporting Items for Systematic Reviews and Meta-Analyses (PRISMA) guidelines (Page et al., 2021) and has been registered on the international prospective register of systematic reviews PROSPERO (registration number: CRD42023473393) under the title “Effects of Exercise Interventions on Memory in Depression: A Three-Level Meta-Analysis”.

## Search Strategy

The search strategy involved computer-based retrieval from CNKI (China National Knowledge Internet), Wanfang, China Biomedicine, PubMed, EMBASE, the Cochrane Library, and Web of Science for randomized controlled trials on exercise improving memory in patients with depression. The retrieval period spanned from the establishment of each database to July 18, 2024. The search strategy used both subject terms and free-text terms, combined with the Boolean operators “and” and “or” for connection.

The keywords include: “depression, depressive disorder, depressive symptom, exercise, aerobic exercise, Taichi, Baduanjin, yoga, cognitive performance, executive function, inhibition, working memory, randomized controlled trial, an so on”. Four English databases were searched, and the Chinese key words “ 运动 (exercise), 有氧运动 (aerobic exercise), 力量训练 (strength training), 太极拳 (Taichi), 八段锦 (Baduanjin), 认知功能 (cognitive function), 执行功能 (executive function), 工作记忆 (working memory), 记忆 (memory) and so on” were searched in three Chinese databases. At the same time, the references to relevant literature were traced (see Table 1).

**Table 1:** Retrieval strategies of each database.

Database	Retrieval strategy
Cochrane And Pubmed	#1“Exercise”[Mesh] OR “Aerobic exercise” [Title/Abstract] OR “Resistance exercise”[Title/Abstract] OR “High-intensity interval” [Title/Abstract] OR “Yoga” [Title/Abstract] OR “Dance” [Title/Abstract] OR “Taichi” [Title/Abstract] OR “Baduanjin” [Title/Abstract] OR “Wuqinxi” [Title/Abstract] OR “Yijinjing” [Title/Abstract] OR “Walking” [Title/Abstract] OR “Physical and mental exercise” [Title/Abstract] #2“Depression”[Mesh] OR “Depressive disorder”[Title/Abstract] OR “Depressive symptom” [Title/Abstract] OR “Emotional depression” [Title/Abstract] OR “Depressive neurosis” [Title/Abstract] OR “Endogenous depression” [Title/Abstract] OR “Deurotic depression” [Title/Abstract] OR “Unipolar depression” [Title/Abstract] #3“Memory” [Mesh] OR “Cognition” [Title/Abstract] OR “Cognitive performance” [Title/Abstract] OR “Cognitive” [Title/Abstract] OR “Working memory” [Title/Abstract]OR #4Randomized controlled trial[Publication Type] OR “Randomized” [Title/Abstract] OR “controlled”[Title/Abstract] OR “Trial” [Title/Abstract] #5 #1 AND #2 AND #3 AND #4
Embase	#1 “Exercise”[exp] OR “Aerobic exercise”[ab, ti] OR “Resistance exercise”[ab, ti] OR “High-intensity interval” [ab, ti] OR “Yoga” [ab, ti] OR “Dance” [ab, ti] OR “Taichi” [ab, ti] OR “Baduanjin” [ab, ti] OR “Wuqinxi” [ab, ti] OR “Yijinjing” [ab, ti] OR “Walking” [ab, ti] OR “Physical and mental exercise” [ab, ti] #2 “Depression”[exp] OR “Depressive disorder” [ab, ti] OR “Depressive symptom” [ab, ti] OR “Emotional depression” [ab, ti] OR “Depressive neurosis” [ab, ti] OR “Endogenous depression” [ab, ti] OR “Deurotic depression” [ab, ti] OR “Unipolar depression” [ab, ti] #3 “Memory” [exp] OR “Cognition” [ab, ti] OR “Cognitive performance” [ab, ti] OR “Cognitive” [ab, ti] OR “Working memory” [ab, ti] OR OR “Cognitive” [ab, ti] #4 “Randomized controlled trial” [exp] OR “Randomized” [ab, ti] OR “Controlled” [ab, ti] OR “Trial” [ab, ti] #5 #1 AND #2 AND #3 AND #4
Web of Science	#1 TS=(“Exercise” OR “Aerobic exercise” OR “Resistance exercise” OR “High-intensity interval” OR “Yoga” OR “Dance” OR “Taichi” OR “Baduanjin” OR “Wuqinxi” OR “Yijinjing” OR “Walking” OR “Physical and mental exercise”) #2 TS=(“Depression” OR “Depressive disorder” OR “Depressive symptom” OR “Emotional depression” OR “Depressive neurosis” OR “Endogenous depression” OR “Deurotic depression” OR “Unipolar depression”) #3 TS=(“Cognition” OR “Cognitive performance” OR “Executive function” OR “Working memory” OR “Memory”) #4 TS=(“Randomized controlled trial” OR “Randomized” OR “Controlled” OR “Trial”) #5 #1 AND #2 AND #3 AND #4
CNKI	(运动 Exercise + 有氧运动 Aerobic Exercise + 抗阻训练 Resistance Training + 力量训练 Power Training + 太极拳 Tai Chi + 瑜伽 Yoga + 八段锦 Baduanjin + 五禽戏 Wuqinxi + 慢跑 Jogging + 快走 Speed Walking) AND (抑郁症 Depression disease + 抑郁 Depression) AND (记忆 Memory + 工作记忆 Working Memory + 认知 Cognition+ 执行功能 Executive function)
Wanfang AND China Biomedicine	(运动 Exercise OR 有氧运动 Aerobic Exercise OR 抗阻训练 Resistance Training OR 力量训练 Power training OR 太极拳 Tai Chi OR 瑜伽 Yoga OR 慢跑 Baduanjin OR 快走 Wuqinxi OR 抑郁症 Jogging OR 抑郁 Speed Walking) AND (抑郁症 Depression disease OR 抑郁 Depression) AND (记忆 Memory OR 工作记忆 Working Memory OR 认知 Cognition OR 执行功能 Executive function)

## Inclusion and Exclusion Criteria

### Inclusion criteria

Participants were patients with depression who met either the diagnostic criteria of the International Classification of Disease (ICD) or the criteria of the Diagnostic and Statistical Manual of Mental Disorders (DSM) and had no other mental diseases. The intervention for the experimental group consisted of either exercise alone or exercise combined with the components of the control group intervention. The minimum duration of the exercise intervention was set at three weeks, which aligns with the average hospitalization period (approximately three to four weeks) for patients receiving inpatient depression treatment. This threshold was chosen for pragmatic reasons, reflecting typical clinical practice rather than being empirically determined. The intervention contents of the control group were irregular exercise or conventional treatment, including drug therapy, occupational therapy, stretching exercise, health education, placebo, etc; The outcome index was memory memory-related index; The type of study was a randomized controlled trial.

### Exclusion criteria

The subjects were non-depressed patients. The languages used in the study were not English or Chinese. Articles in which data could not be obtained or extracted for estimating effect size even after contacting the authors. Studies using a single bout of exercise as an intervention. Studies using animal models, conference abstracts, book chapters or reviews.

## Literature Screening and Data Extraction

### Literature screening

Two researchers (YN Z and XL Z) independently screened the literature according to the inclusion and exclusion criteria. First, the retrieved literature was imported into Endnote X9 to eliminate duplicate literature and read the titles and abstracts of literature for preliminary screening. Secondly, the full text was screened again to ensure that the content of the study and the outcome indicators met the inclusion criteria. Then, the final included literature was determined. If two researchers had disagreements, a third researcher (C L) joined in the discussion to make the final decision.

### Data extraction and coding strategy

Two authors (YN Z and XL Z) extracted data separately by using a pre-developed extraction form in Microsoft Excel. The agreement of extraction between the two was 96.82%. The extracted information included basic information (author, year of publication, country, age, sample size), experimental characteristics (two groups of intervention content and exercise intensity), and all memory-related outcome indicators in each literature. The outcome data were collected during pre-intervention, during-intervention and post-intervention. If the data was missing or unclear, the original author would be contacted through email; when the information extracted by two researchers (YN Z and XL Z) was inconsistent, a third researcher (C L) would join in the discussion and make a decision together.

Due to the lack of commonality in exercise frequency, we could not perform subgroup analyses based on this variable; therefore, we coded only exercise type, duration, intensity, intervention content of the experimental group, and age. We categorized exercise type as aerobic, strength, and mind-body exercise; coded exercise time as short (≤60 min) and long (>60 min); classified exercise duration as ≤12 weeks and >12 weeks; and defined exercise intensity as low-to-moderate, moderate, and moderate-to-vigorous. (Ren et al., 2023; Ren et al., 2024). Additionally, we coded the intervention content of the experimental group as either exercise alone or exercise combined with other therapies. Age was categorized into young adults (18–44 years), middle-aged adults (45–64 years), and older adults (≥ 65 years).

### Assessment of study quality

Two reviewers (YN Z and XL Z) independently assessed the risk of bias in the included studies using the Cochrane Risk of Bias 2 (RoB 2) tool. RoB 2 was selected instead of the PEDro scale because it represents the current Cochrane-recommended standard for randomized trials and is more appropriate for exercise interventions with psychological or cognitive outcomes, where items such as participant or therapist blinding in PEDro are often infeasible and may artificially deflate quality scores. This modification was also made in response to prior reviewer feedback, and the review team agreed that RoB 2 would provide a more rigorous and transparent assessment framework. RoB 2 comprises five domains: randomization process; deviations from intended interventions; missing outcome data; measurement of the outcome; and selection of the reported result (Sterne et al., 2019). Each domain is rated as low risk of bias, some concerns, or high risk of bias. An overall judgment of low risk of bias is assigned when all domains are rated low risk; some concerns is assigned when at least one domain is rated some concerns and none is rated high risk; and high risk of bias is assigned if any single domain is rated high risk (Sterne et al., 2019; Wang et al., 2025). In case of disagreement, a third researcher (C L) would discuss and make the final decision.

GRADEpro software was used to evaluate the quality of outcome evidence. From “high risk of bias in study design or implementation”, “high heterogeneity or inconsistency of results”, “indirectness of evidence”, “imprecision of results” and “high probability of publication bias” were used to evaluate downgrade or upgrade. Ultimately, each level of evidence was rated as “high”, “moderate”, “low” and “very low”(Li et al., 2023).

### Statistical analysis

This study utilized the metafor package in R version 4.3.0 to conduct a three-level meta-analysis using a random effects model (Assink & Wibbelink, 2016; Cui et al., 2024; Viechtbauer, 2010). This model considers the following three categories of effect size (ES) variability: sampling variance (level 1); within-study variance (level 2); and between-study variance (level 3) (Cheung, 2014). With the use of the restricted maximum-likelihood estimation (REML) method, pooled ES of exercise on memory was determined using Hedges’ g and 95% confidence intervals (CI). When the ES was divided according to Hedges’ g, *P* < 0.05 was statistically significant. Hedges’ g<0.20 refers to a small effect, 0.20–0.49 is a small-to-moderate effect, 0.50–0.79 is a medium effect, and ≥ 0.80 is a large effect (Cohen, 1992). 95% prediction intervals (PI) were calculated to determine the expected range in which an ES in future identical studies will fall.

Testing for statistical significance in level 2 (within-study) and level 3 (between-study) variances were performed using likelihood ratio tests (LRT).The I^2^ statistic was used to estimate of total between-study heterogeneity (1–49% indicating low, 50–74% indicating moderate, and 75–100% indicating high) (Higgins et al., 2003). To evaluate the robustness of the findings, we conducted a series of additional sensitivity analyses following recommended meta-analytic practices. Publication bias was assessed using Egger’s regression test and a contour-enhanced funnel plot (Egger et al., 1997). Influence diagnostics (including studentized residuals, Cook’s distances, DFFITS, covariance ratios, hat values, and QE deletion diagnostics) were computed to identify potential influential or outlying effect sizes (Viechtbauer & Cheung, 2010). Leave-one-out analyses were performed to test the stability of the pooled effect. Based on prior evidence, exercise intensity was designated a priori as the primary moderator (Liu et al., 2024). The other moderators (duration, intervention content, age, intervention type, session time) were considered exploratory. For the exploratory tests, we controlled multiplicity using the Benjamini–Hochberg false discovery rate (FDR, *q* = 0.05) and report both unadjusted omnibus P and FDR-adjusted q values (Benjamini & Hochberg, 1995).

## Results

### Literature search results

**Figure 1:**
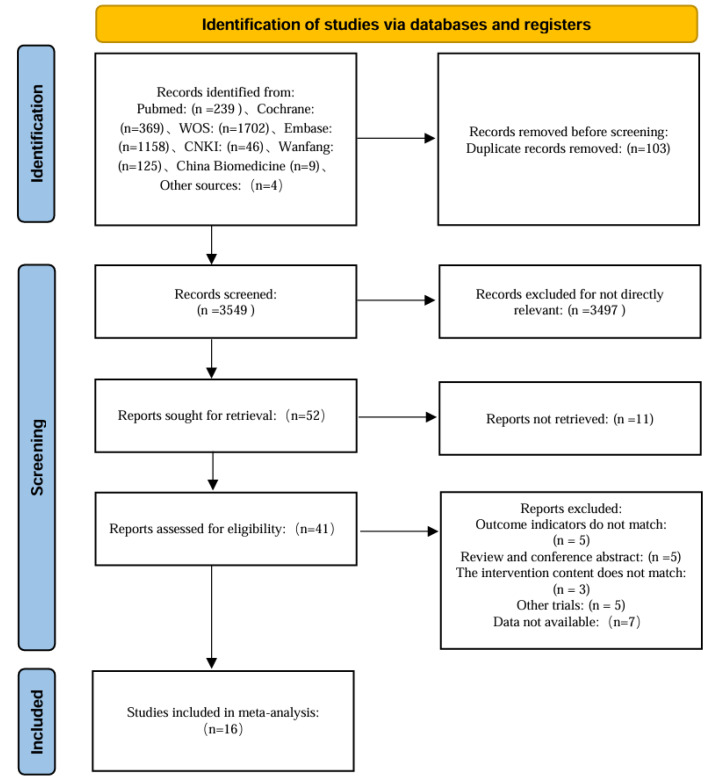
Flow chart of literature screening.

Seven databases yielded 3,648 records; citation chasing added four (total 3,652). EndNote X9 de-duplication removed 103, leaving 3,549 for title/abstract screening. Of 52 full texts sought, 11 were unobtainable; 41 were assessed, and 25 were excluded (outcome mismatch, five; review/abstract, five; intervention ineligible, three; non-randomized controlled trial (RCT), three; data not extractable, seven; other, (2). Sixteen studies remained (14 English, two Chinese; including one grey source) (see Fig. 1).

### Characteristics of the included literature

The 16 studies were published between 2001 and 2022, originating from Germany (two), the USA (four), Switzerland (one), Denmark (three), India (two), and China (four). The age of patients with depression ranged from 31.50 to 70.55 years. The intervention contents of the experimental group were aerobic exercise, strength training, Taichi, aerobic exercise combined with conventional rehabilitation, and yoga combined with conventional rehabilitation, whereas the intervention contents of the control group were occupational or artistic therapy, placebo, etc; Exercise intensity was divided into low-to-moderate intensity, medium intensity and medium-to-vigorous intensity. The outcome indicators were DSTB(Digit span Test backward), VIMTLT (Verbaler Lern-und Merkfähigkeitstest learning, total) and so on, as shown in Table 2.

**Table 2:** Basic characteristics of the included literature. HRR for Heart rate reserves. HRmax for Maximal heart rate. RM for repetition maximum. d/wk for day/week. wk for week. CLVT for The California Verbal Learning Test II. RAVLTTS for Rey auditory verbal learning test total score. RAVLTDLB for Rey auditory verbal learning test-distraction list B. RAVLTIR for Rey auditory verbal learning test-immediate recall. RAVLTIRDR for Rey auditory verbal learning test-delayed recall. DSTF for Digit span Test Forward. DSTB for Digit span Test backward. ROCFT for 30-minute Delayed Recall. WMSLMIR for Wechsler Memory Scale Logical Memory Immediate Recall. WMSLMDR for Wechsler Memory Scale Logical Memory Delayed Recall. WMSVRIR for Wechsler Memory Scale Visual Reproduction Immediate Recall. WMSVRDR for Wechsler Memory Scale Visual Reproduction Delayed Recall. WMSLM for Wechsler Memory Scale Logical memory. WMSVPE for Wechsler Memory Scale Verbal pairs, easy. WMSVPH for Wechsler Memory Scale Verbal pairs, hard. DS for Digit Span. BSRT for Buschke Selective Reminding Test. RCFT for Rey Complex Figure Test. WMSRSTM for Wechsler Memory Scale-Revised short-term memory. VLMTLT for Verbaler Lern-und Merkfähigkeitstest learning, total. VLMTLT for Verbaler Lern-und Merkfähigkeitstest recall. VRS for Visual Reproduction Subtest. TMS for Tower of London- total move score.

Included literature	Country	Number of sample (E/C)	Age	Intervention content		Intensity	Outcome measure
				Experimental group	Control group		
Buschert et al. (2019)	Germany	18/20	47.37	Aerobic exercise, 30 min, 2–3 d/wk, 3–4 wk	Occupational or art therapy	85%HRmax/ Moderate-to- Vigorous	DSTB, VLMTLT, VLMTRE, WMSRSTM
Hoffman et al. (2008)	USA	104/49	51.70	Aerobic exercise, 45 min, 3 d/wk, 16 wk	Placebo	70–85%HRR/ Moderate-to- Vigorous	WMSLM, DSTF, WMSVPE, DSTB, Ruff
Imboden et al. (2020)	Switzerland	22/20	39.90	Aerobic exercise with standard inpatient treatment, 45 min, 3 d/wk, 6 wk	Stretching with standard inpatient treatment	60–75%HRmax/ Moderate	sequence of numbers
Khatri et al. (2001)	USA	42/42	56.73	Aerobic exercise, 45 min, 3 d/wk, 16 wk	Medication	70–85%HRR/ Moderate-to- Vigorous	WMSLMIR, WMSLMDR, WMSVRIR, DSTB, WMSVRDR, DSTF,
Krogh et al. (2009)	Denmark	47/48/42	38.90	Aerobic exercise, Strength training, 90 min, 2 d/wk, 16 wk	Relaxation	50–75%RM, 70–89%HRmax/ Moderate-to- Vigorous	DS, BSRT
Lavretsky et al. (2011)	USA	36/37	70.55	Taichi combine with medication, 120 min, 1 d/wk, 10 wk	Health education with medication	Low-to-Moderate	CVLT
Lavretsky et al. (2022)	USA	62/63	69.30	Taichi, 60 min, 1 d/wk, 12 wk	Health education	Low-to-Moderate	ROCFT
Sharma et al. (2006)	India	15/15	31.77	Yoga combined with conventional rehabilitation, 30 min, d/wk, 8 wk	Conventional therapy	Low-to-Moderate	DSTB, DSTF
Zhang & Chen (2019)	China	22/20	31.80	Aerobic exercise combine with medication, 3 km, 8 wk	Medication	170-age/ Moderate	DSTB, DSTF, VRS
Zhang et al. (2022)	China	20/19	50.68	Taichi, 90 min, 2 d/wk, 12 wk	Daily life	Low-to-Moderate	TMS
Krogh et al. (2012)	Denmark	56/59	41.55	Aerobic exercise, 45 min, 3 d/wk, 12 wk	Stretching	65–80%VO_2_max/ Moderate-to- Vigorous	BSRT, DSTB, DSTF, RCFT
Krogh et al. (2014)	Denmark	41/38	41.30	Aerobic exercise, 45 min, 3 d/wk, 12 wk	Stretching	80%HRmax/ Moderate-to-Vigorous	BSRT, RCFT
Chen et al. (2021)	China	63/32	31.50	Aerobic exercise combined with medication, 30-60 min, 3 d/wk, 16 wk	Medication	64–76%HRmax/ Moderate	DSTB, DSTF, VRS
Zheng et al. (2019)	China	30/30	72.67	Aerobic exercise combined with conventional rehabilitation, 60 min, 5 d/wk, 4 wk	Conventional therapy	Moderate	Immediate recall, Delay recall, DS
Halappa et al. (2018)	India	16/26/23	33.72	Yoga only/Yoga with medication, 60 min, 12wk	Medication	Low-to-Moderate	RAVLTTS, RAVLTDLB, RAVLTIR, RAVLTIRDR, DSTF, DSTB
							
Oertel-Knöchel et al. (2014)	Germany	6/8	40.00	Aerobic exercise, 45 min, 3 d/wk, 4 wk	Standardized Therapeutic Routines	60–70% HRmax/Moderate	Verbal learning, Visual learning

### Risk of bias in the included studies

**Figure 2:**
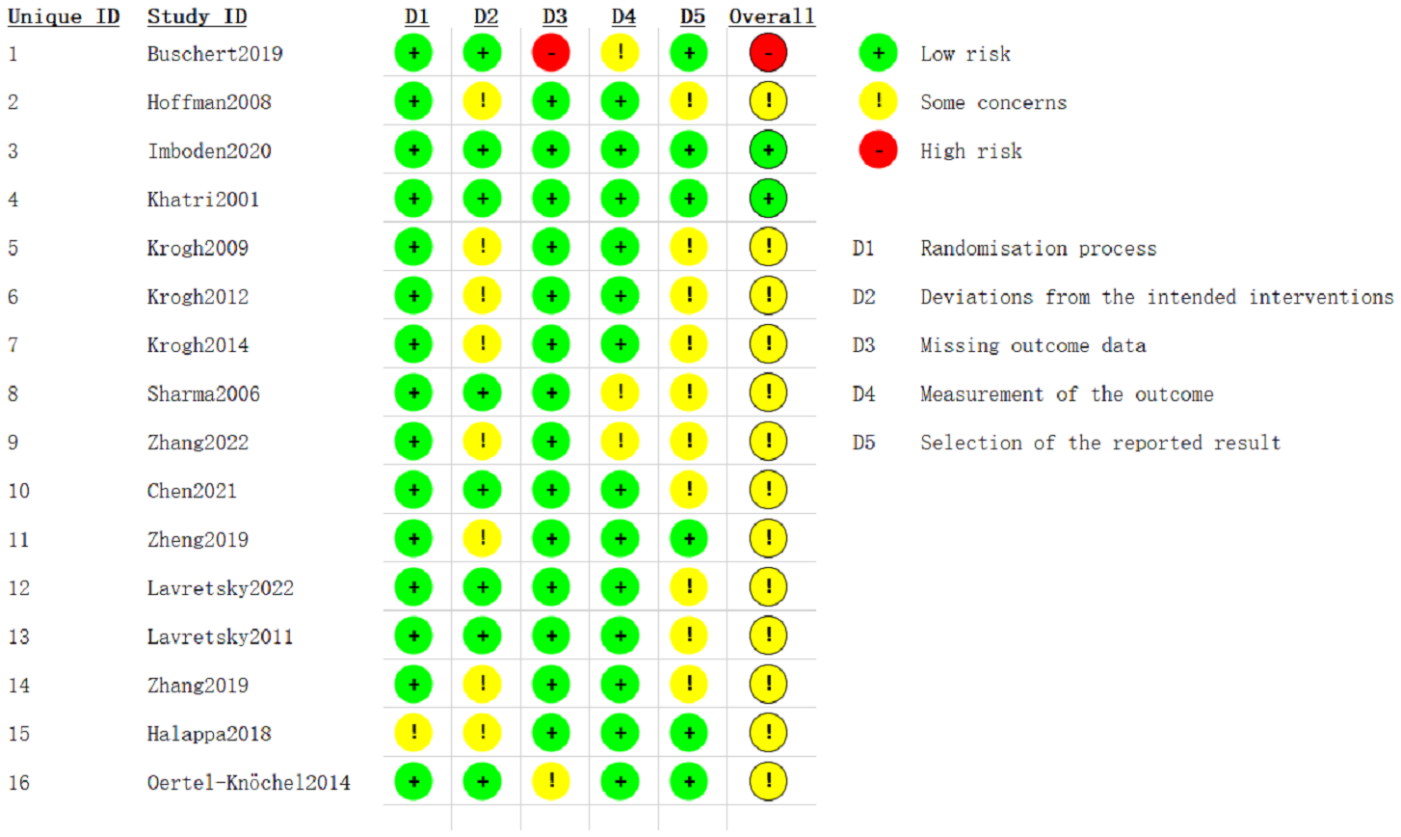
RoB 2 risk-of-bias assessment. Studies: Buschert et al. (2019), Hoffman et al. (2008), Imboden et al. (2020), Khatri et al. (2001), Krogh et al. (2009), Lavretsky et al. (2011), Lavretsky et al. (2022), Sharma et al. (2006), Zhang & Chen (2019), Zhang et al. (2022), Krogh et al. (2012), Krogh et al. (2014), Chen et al. (2021), Zheng et al. (2019), Halappa et al. (2018), Oertel-Knöchel et al. (2014).

Risk of bias was assessed for the included studies using the RoB 2 tool. Across the five domains, 15 studies were judged low risk for the randomization process; eight were low risk for deviations from intended interventions; 14 were low risk for missing outcome data; 13 were low risk for measurement of the outcome; and six were low risk for selection of the reported result. Overall, one study was rated high risk of bias, two were low risk of bias, and 13 raised some concerns (Fig. 2).

### The interventional effects of exercise on memory in patients with depression

**Figure 3:**
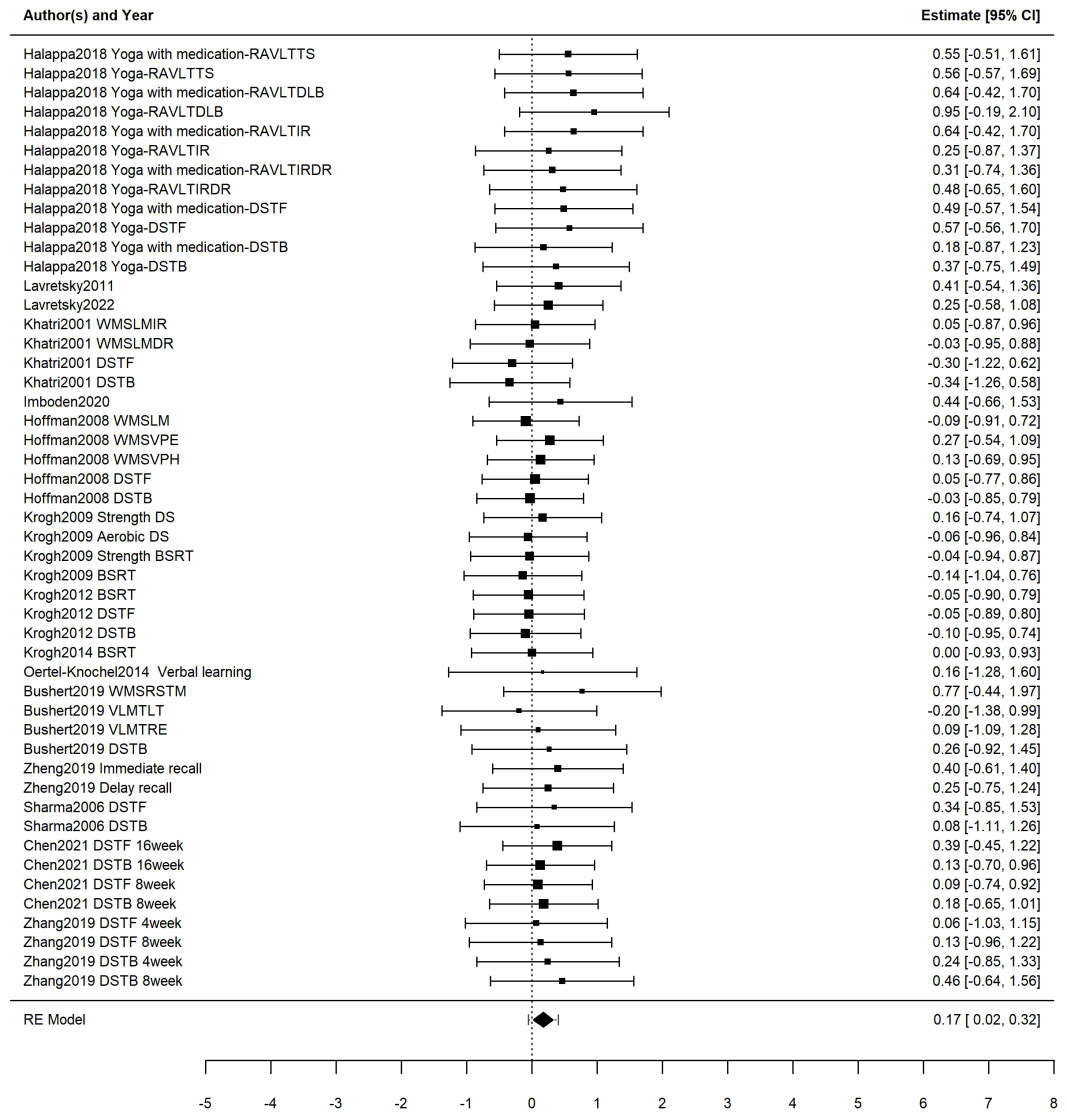
Forest plot illustrating the effects of exercise on verbal memory in patients with depression. Studies: Buschert et al. (2019), Hoffman et al. (2008), Imboden et al. (2020), Khatri et al. (2001), Krogh et al. (2009), Lavretsky et al. (2011), Lavretsky et al. (2022), Sharma et al. (2006), Zhang & Chen (2019), Zhang et al. (2022), Krogh et al. (2012), Krogh et al. (2014), Chen et al. (2021), Zheng et al. (2019), Halappa et al. (2018), Oertel-Knöchel et al. (2014).

We employed random-effects models to aggregate effect sizes. For verbal memory, the three-level random-effects model showed a small, statistically significant effect of exercise (*g* = 0.17, 95% CI [0.02–0.32], *p* = 0.03), with a 95% PI of −0.06 to 0.40, as shown in Fig. 3. The two-level random-effects model yielded a similar result (*g* = 0.17, 95% CI [0.03–0.32], *p* = 0.02), with a 95% PI of 0.03 to 0.32, indicating a likely minimal in magnitude but consistent benefit. Heterogeneity was low (*Q* = 13.59, *p* > 0.05).

**Figure 4:**
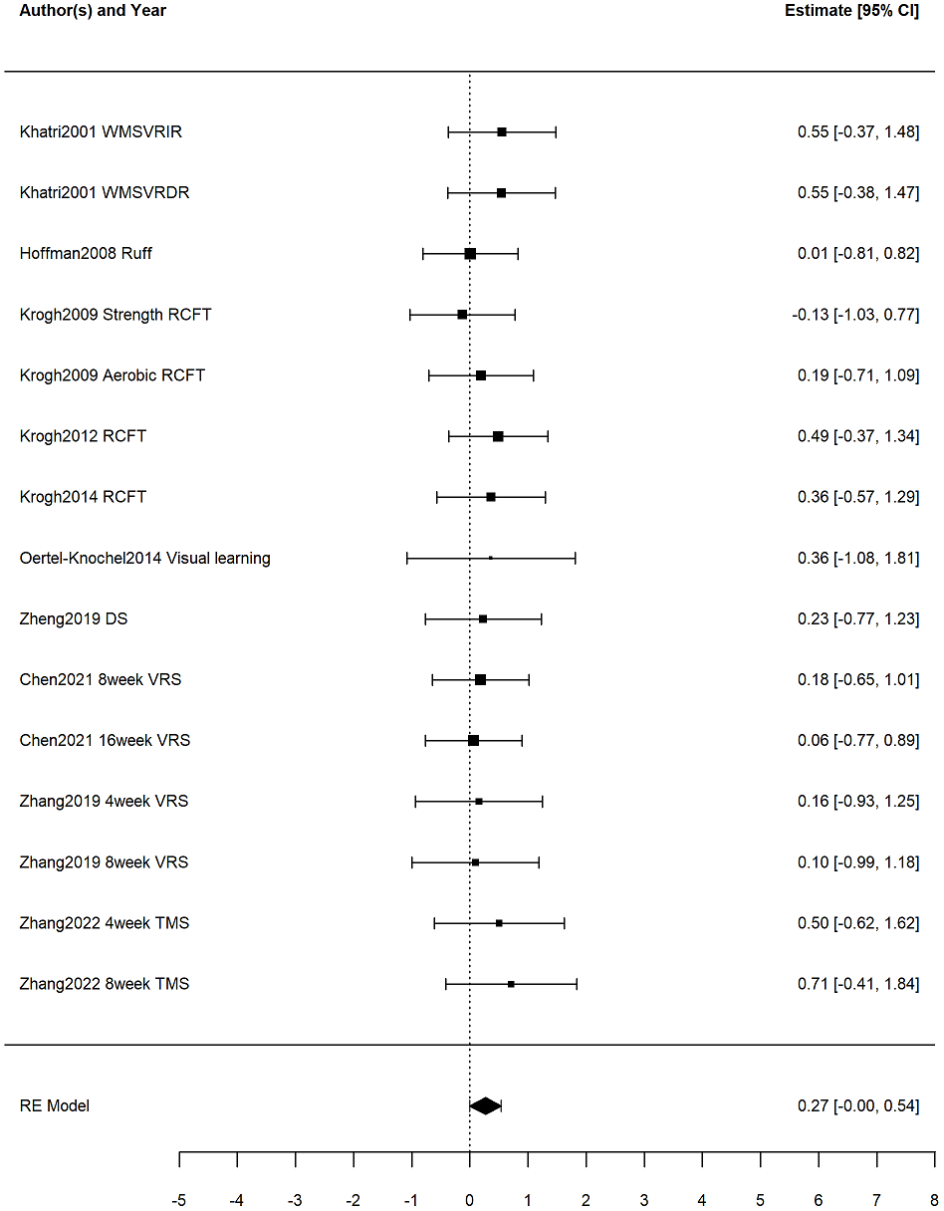
Forest plot illustrating the effects of exercise on visual memory in patients with depression. Studies: Buschert et al. (2019), Hoffman et al. (2008), Imboden et al. (2020), Khatri et al. (2001), Krogh et al. (2009), Lavretsky et al. (2011), Lavretsky et al. (2022), Sharma et al. (2006), Zhang & Chen (2019), Zhang et al. (2022), Krogh et al. (2012), Krogh et al. (2014), Chen et al. (2021), Zheng et al. (2019), Halappa et al. (2018), Oertel-Knöchel et al. (2014).

For visual memory, the three-level random-effects model indicated no statistically significant effect (*g* = 0.27, 95% CI [−0.00 to −0.54], *p* = 0.05), with a PI spanning the null (−0.00 to 0.54) (Fig. 4). The two-level random-effects model produced nearly identical estimates (*g* = 0.27, 95% CI [−0.00–0.54], *p* = 0.05), with a PI spanning the null (−0.00 to 0.54), reinforcing the conclusion that any effect, if present, is likely minimal and uncertain. Heterogeneity was again low (*Q* = 3.37, *p* > 0.05).

### Influence analysis

**Figure 5:**
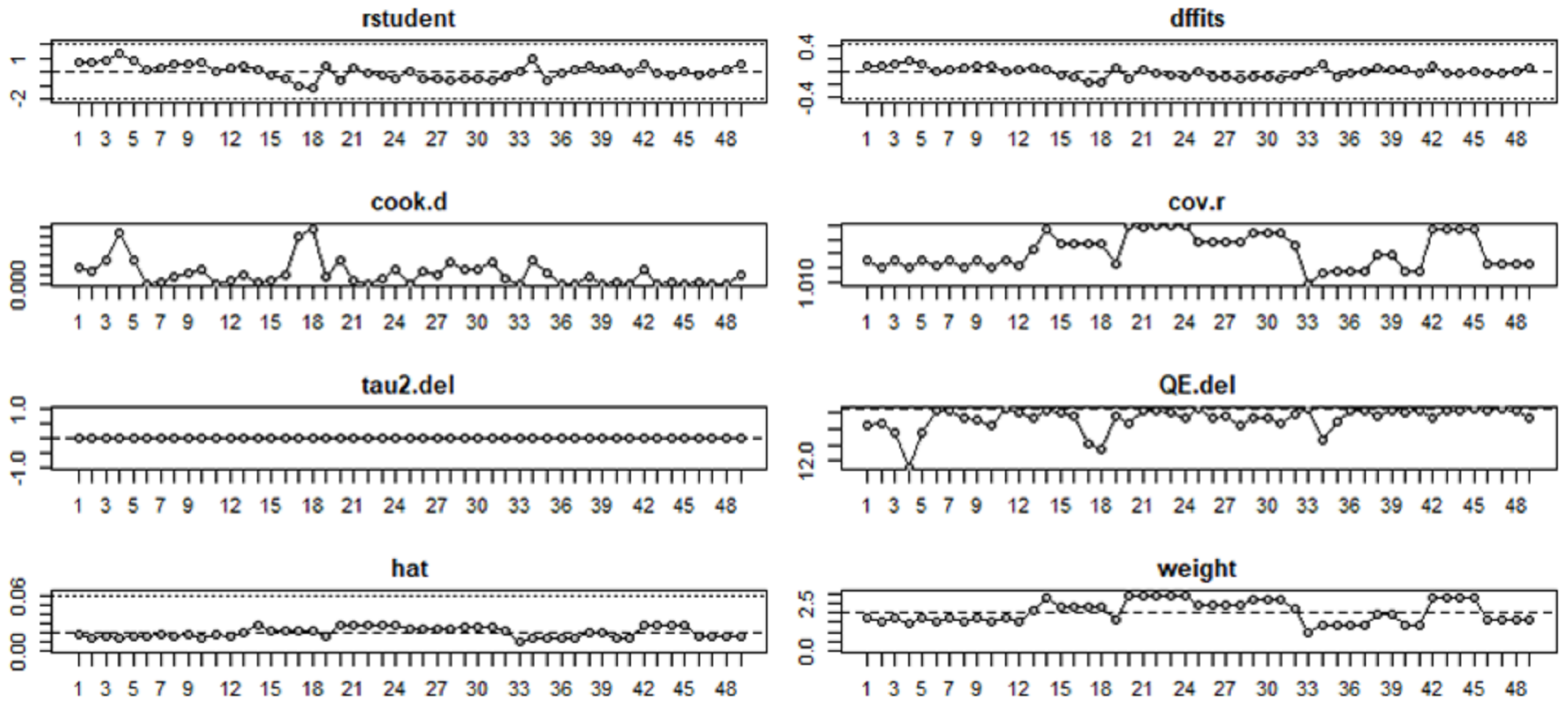
Influence analysis of the effects of exercise on verbal memory in patients with depression.

**Figure 6:**
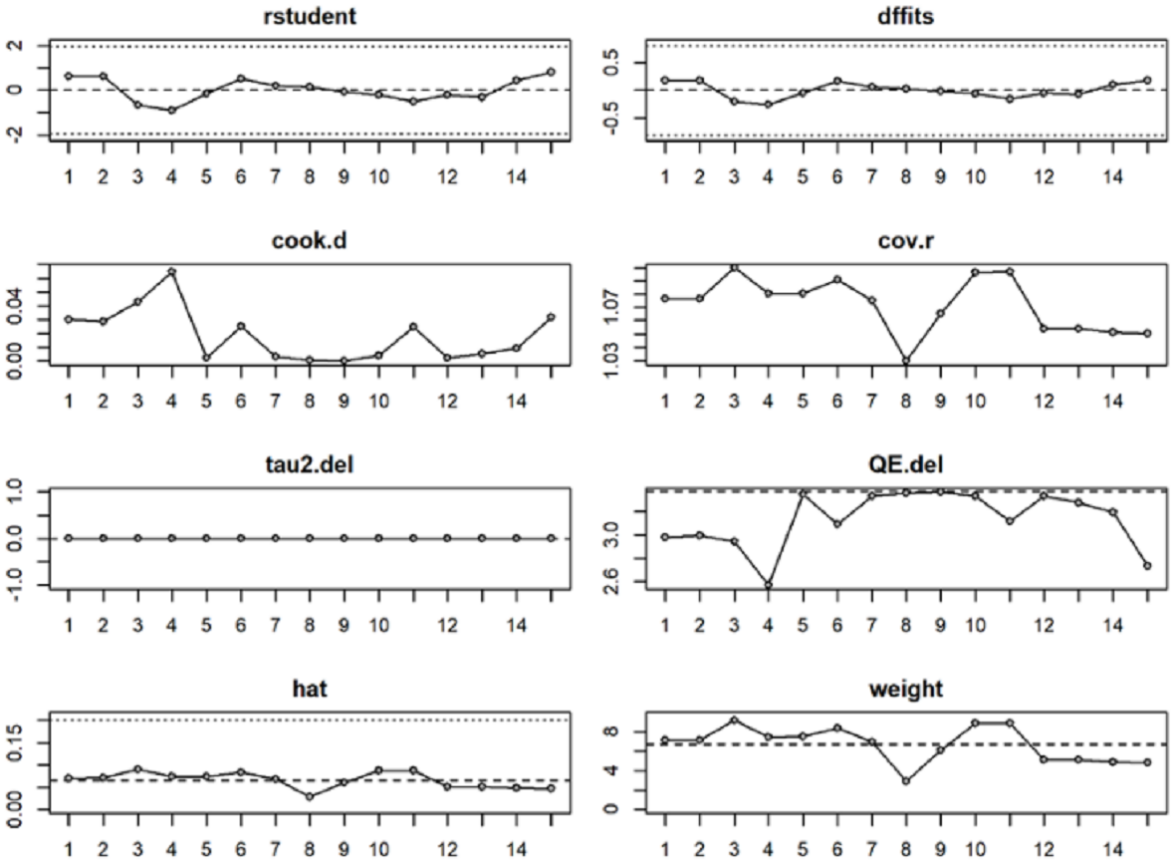
Influence analysis of the effects of exercise on visual memory in patients with depression.

We conducted formal influence diagnostics (studentized residuals, Cook’s distance, hat values, and DFBETAs) and complemented them with a leave-one-study-out analysis. For verbal memory, deleting any single study yielded pooled estimates of *g* = 0.16–0.19 with *p* = 0.02–0.04, leaving the direction and statistical significance unchanged. For visual memory, the pooled estimate ranged *g* = 0.24–0.30 with *p* = 0.04–0.08; although the direction remained positive, the *p*-values straddled 0.05, indicating marginal and non-robust evidence. Influence diagnostics did not identify any outliers affecting either outcome. This pattern is illustrated in Figs. 5 and 6 and is further supported by the data presented in Tables S1 and S2.

### Heterogeneity analysis

In the analysis of verbal memory, the log-likelihood ratio test indicated that the between-study variance (level 3) was not significant, *χ*^2^ = 0.17, *p* = 0.34, suggesting that the three-level model did not outperform the two-level model. The within-study variance (level 2) was also not significant, *χ*^2^ = 0.00, *p* = 0.50, further indicating that the three-level model did not provide a superior fit compared to the two-level model. Despite this, employing the three-level model remains meaningful. In terms of overall variance, the within-study variance (level 2) accounted for 8.60%, and the between-study variance (level 3) accounted for 2.87%.

In the analysis of visual memory, the log-likelihood ratio test revealed that the between-study variance (level 3) was not significant, *χ*^2^ = 0.00, *p* = 0.50, indicating that the three-level model did not outperform the two-level model. The within-study variance (level 2) was also not significant, *χ*^2^ = 0.00, *p* = 0.50, further suggesting that the three-level model did not provide a superior fit compared to the two-level model. Nevertheless, the use of the three-level model remains meaningful. In terms of overall variance, the within-study variance (level 2) accounted for 1.25%, and the between-study variance (level 3) accounted for 9.66%.

Although the three-level model did not significantly improve fit, we retained it because it reflects the hierarchical data-generating process with multiple outcomes nested within studies and appropriately accounts for within-study dependence; moreover, variance-component tests are underpowered when parameters lie on the boundary, so near-zero estimates alone are not sufficient grounds to collapse the hierarchy, and retaining the structure yields valid—and typically more conservative—inference (Van den Noortgate et al., 2013).

### Moderator analysis

Since exercise did not affect visual memory in patients with depression, we examined moderators only for verbal memory. Exercise intensity was pre-specified as the primary moderator, whereas exercise duration, type, session time, intervention content of the experimental group, and age were treated as exploratory; for exploratory moderators, multiplicity was controlled using the Benjamini–Hochberg FDR, as shown in Table 3.

**Table 3:** Effects of moderating factors on verbal memory in patients with depression.

	K, N	g	95% CI	F	DF	P, q (FDR)	Level 2 vaniance	Level 3 vaniance
Duration of exercise				3.04	1	0.09, 0.24	1.22%	4.94%
≤12 week	11,758	0.27	0.09, 0.46			<0.01		
>12 week	4,499	0.02	−0.21, 0.25			0.87		
Intervention content of the experimental group				2.20	1	0.14, 0.24	<1.00%	0.11%
Only exercise	9,776	0.09	−0.09, 0.27			0.32		
Exercise combined with other therapies	6,379	0.31	0.08, 0.54			0.01		
Age				0.87	2	0.43, 0.53	<1.00%	1.69%
Young adults (18–44 years)	8,607	0.22	0.03, 0.41			0.03		
Middle adults (45–64 years)	3,267	0.03	−0.25, 0.30			0.85		
Old adults (≥65 years)	3,258	0.32	−0.17, 0.81			0.19		
Intervention type				2.48	2	0.10, 0.24	2.91%	1.81%
Aerobic exercise	10, 792	0.08	−0.09, 0.25			0.36		
Strength exercise	1,89	0.06	−0.59, 0.72			0.85		
Mind-body exercise	4,293	0.43	0.16, 0.71			<0.01		
Session time of exercise				0.25	1	0.62, 0.62	<1.00%	3.23%
≤60 min	12,922	0.18	0.02, 0.35			0.03		
>60 min	2,210	0.07	−0.38, 0.51			0.77		
Intensity				3.39	2	0.04	2.36%	9.56%
Low-to-moderate	4,293	0.43	0.16, 0.71			<0.01		
only moderate	4,241	0.24	−0.05, 0.53			0.11		
Moderate-to-vigorous	6,598	0.00	−0.20, 0.20			>0.49		

Exercise duration was not a statistically significant moderator (*F* = 3.04, *p* = 0.09, *q* = 0.24). Shorter interventions (≤12 weeks) showed a small, statistically significant effect (*g* = 0.27, 95% CI [0.09–0.46], *p* < 0.01), whereas longer interventions did not (*g* = 0.02, 95% CI [−0.21–0.25], *p* = 0.87). This suggests that any benefits may emerge relatively early and may not increase with longer treatment durations.

Exercise type was also not a significant moderator (*F* = 2.48, *p* = 0.10, *q* = 0.24). Neither aerobic exercise (*g* = 0.08, 95% CI [0.09–0.25], *p* = 0.36) nor strength training (*g* = 0.06, 95% CI [−0.59–0.72], *p* = 0.85) demonstrated significant effects. However, mind–body exercise yielded a small-to-moderate and statistically significant effect (*g* = 0.43, 95% CI [0.16–0.71], *p* < 0.01), indicating a potentially meaningful benefit for this exercise modality.

Session time was not a significant moderator (*F* = 0.25, *p* = 0.62, *q* = 0.62). Shorter sessions (≤60 min) produced a small but statistically significant effect (*g* = 0.18, 95% CI [0.02–0.35], *p* = 0.03), whereas longer sessions did not (*g* = 0.07, 95% CI [−0.38–0.51], *p* = 0.77). These findings suggest that briefer sessions may be more feasible and still beneficial.

Exercise intensity emerged as a significant moderator (*F* = 3.39, *p* = 0.04). Low-to-moderate intensity produced a small-to-moderate, statistically significant effect (*g* = 0.43, 95% CI [0.16–0.71], *p* < 0.01). Moderate intensity alone did not reach significance (*g* = 0.24, 95% CI [−0.05–0.53], *p* = 0.11), nor did moderate-to-vigorous exercise (*g* = 0.00, 95% CI [−0.20–0.20], *p* > 0.49). This pattern suggests that more strenuous activity may not yield additional cognitive benefits and could even reduce adherence or tolerability.

Intervention content (exercise alone *vs.* combined with other therapies) was not a significant moderator (*F* = 2.20, *p* = 0.14, *q* = 0.24). Exercise alone did not yield significant effects (*g* = 0.09, 95% CI [−0.09–0.27], *p* = 0.32). However, combined interventions produced a small but statistically significant effect (*g* = 0.31, 95% CI [0.09–0.54], *p* = 0.01), suggesting potential synergistic benefits when exercise is integrated with other therapeutic approaches.

Age group was not a statistically significant moderator (*F* = 0.87, *p* = 0.43, *q* = 0.53). Young adults demonstrated a small but statistically significant effect (*g* = 0.22, 95% CI [0.03–0.41], *p* = 0.03), whereas middle-aged adults (*g* = 0.03, 95% CI [−0.25–0.30], *p* = 0.85) and older adults (*g* = 0.32, 95% CI [−0.18–0.81], *p* = 0.19) did not show significant improvements. Given the limited number of studies involving older adults, these results should be interpreted cautiously.

### Publication bias

**Figure 7:**
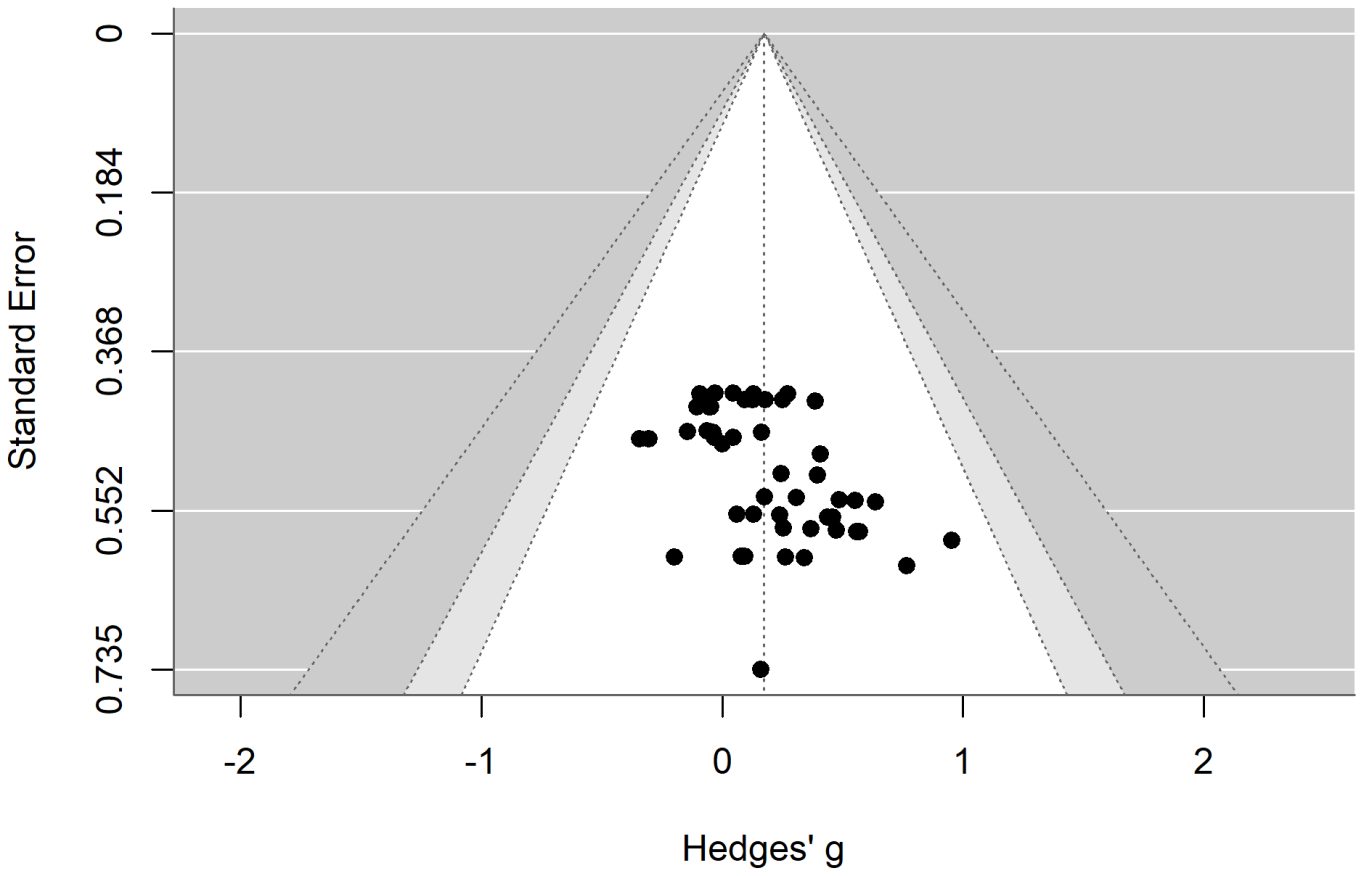
Funnel plot illustrating the effects of exercise on verbal memory in patients with depression.

**Figure 8:**
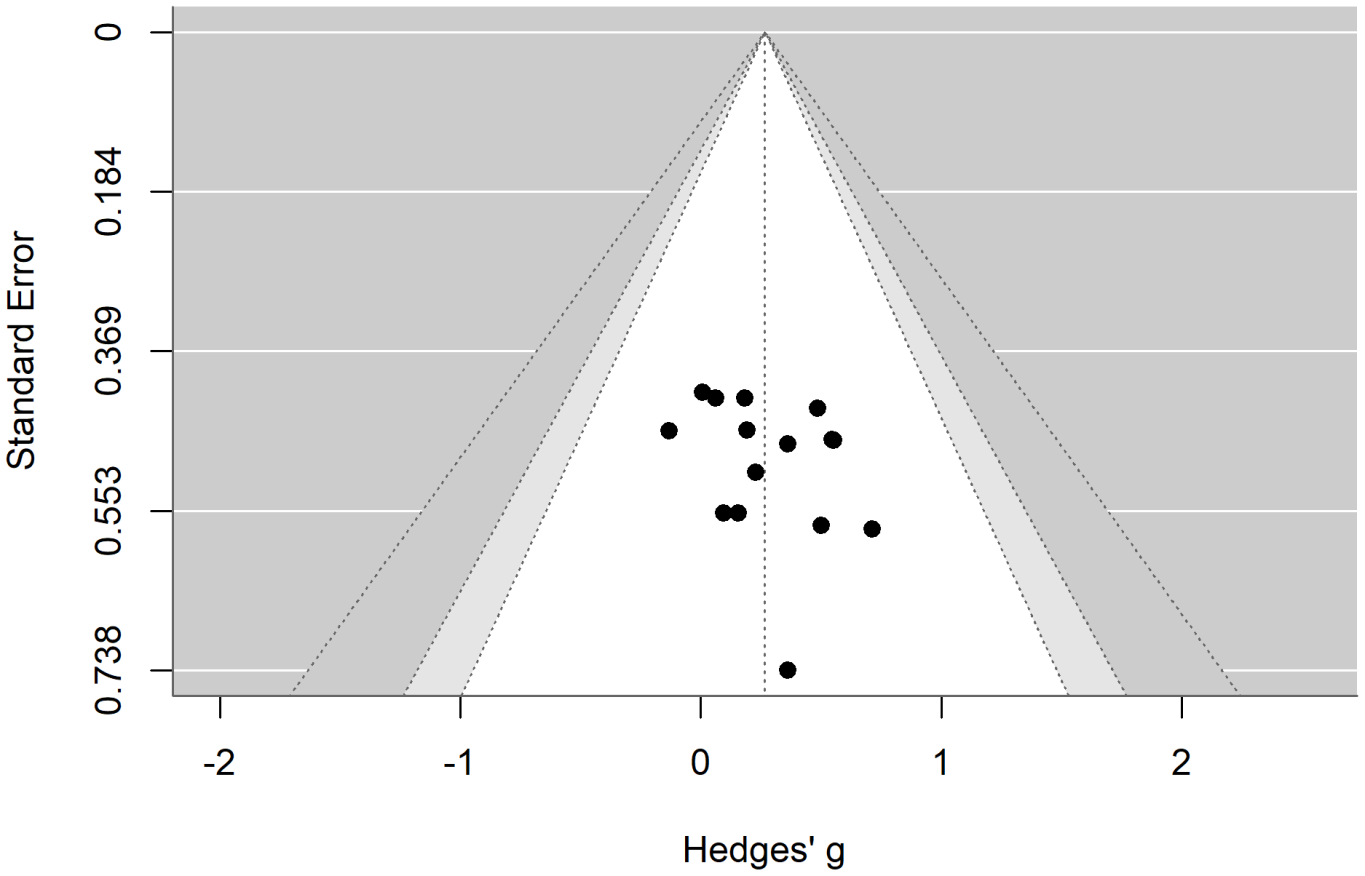
Funnel plot illustrating the effects of exercise on visual memory in patients with depression.

We assessed publication bias using a funnel plot and Egger’s regression test. The results indicated that the funnel plot was largely symmetric. Additionally, Egger’s regression test suggested that there was no publication bias regarding the effects of exercise on verbal memory (*t* = 1.79, *p* = 0.08) and spatial memory (*t* = 0.59, *p* = 0.56) in patients with depression, as shown in Figs. 7 and 8.

### GRADEpro evidence quality assessment

We conducted an evidence-quality assessment for verbal memory and visual memory. Due to the lack of distinction regarding the severity of depression among the included studies, the level of inconsistency was downgraded by one level, resulting in an overall evidence quality rating of moderate (see Table 4).

**Table 4:** Level of evidence for outcome indicators. 1, Study limitation; 2, Inconsistency; 3, Indirectness; 4, Imprecision; 5, Publication bias; D, Downgrade 1 Level; N, Not Downgraded.

Outcome	Evaluation of evidence quality level					Relative effect size	Level
	1	2	3	4	5		
Verbal memory	N	D^*^	N	N	N	0.017, 0.323	Moderate
Visual memory	N	D^*^	N	N	N	−0.003, 0.536	Moderate

**Notes.**

* Some studies did not distinguish the degree of depression in patients with depression.

## Discussion

### Main study findings

This study indicates that exercise can improve verbal memory in patients with depression; however, it does not demonstrate an effect on visual memory. Both findings are classified as moderate evidence, suggesting that future research may modify these conclusions. Although the pooled effect on verbal memory was small (*g* = 0.17) and the 95%PI included zero, such modest effects—particularly when tied to modifiable behavioral factors—may still be clinically relevant. A g-value of 0.17 roughly corresponds to a 4–6% improvement on standardized verbal memory tests, representing a subtle but potentially meaningful enhancement in everyday cognitive functioning.

### The impact of exercise on memory in patients with depression

This study found that exercise can improve verbal memory in patients with depression, whereas no effect was observed for visual memory. Although the effect on verbal memory was small, between-study uncertainty suggests that this benefit may not consistently replicate. In contrast, Sun et al. (2018) conducted a traditional meta-analysis in which effect sizes from individual studies were pre-aggregated (five studies for verbal memory and three studies for visuospatial memory), yielding pooled effects of *g* = 0.09 (*n* = 577) for verbal memory and *g* = 0.35 (*n* = 302) for visuospatial memory; neither reached statistical significance. Similarly, Brondino et al. (2017) aggregated effect sizes from five studies on verbal memory and three studies on visuospatial memory and applied a conventional two-level meta-analytic model. Their findings indicated no improvement in verbal memory (*g* = 0.05, *n* = 577), but a small improvement in visuospatial memory (*g* = 0.24, *n* = 302). Unlike Sun et al. (2018), Brondino et al. (2017) further differentiated the effects of different exercise modalities. This discrepancy may be attributed to our use of a three-level meta-analysis method, which allowed for a comprehensive statistical analysis of all data related to verbal learning and memory as well as visual learning and memory. This approach minimized the issue of information loss that could reduce statistical power and mitigated the potential inflation of results due to correlations between effect sizes. Previous research employing three-level meta-analyses has yielded similar conclusions. Ren et al. (2024) also employed a three-level meta-analysis, incorporating eight studies, and found that aerobic exercise can improve memory in patients with depression. Unfortunately, they did not further differentiate between verbal memory and visual memory in their analysis. By explicitly modeling the dependency structure among multiple outcomes within studies, our three-level model reduced within-study redundancy, prevented disproportionate weighting of studies that reported more outcomes, and yielded more conservative and arguably more robust pooled estimates than would likely have been obtained using a traditional two-level approach with pre-aggregated effect sizes.

Taken together, these methodological features mean that the present synthesis contributes several elements that previous meta-analyses could not capture. First, it provides domain-specific pooled estimates for verbal and visual memory rather than a single global memory outcome, thereby clarifying that observable exercise-related benefits appear to be confined to verbal memory. Second, the three-level structure, combined with an expanded study base (including Chinese databases and gray literature), allowed a more systematic evaluation of potential moderators (*e.g.*, intensity, session duration, exercise type, and intervention format), offering preliminary guidance on which exercise prescriptions may be most promising for targeting memory outcomes in depression.

From a mechanistic perspective, our findings align with current neurobiological frameworks linking exercise to domain-specific cognitive enhancement. Aerobic exercise promotes hippocampal neuroplasticity through increased BDNF expression, neurogenesis, and dentate gyrus remodeling-mechanisms known to preferentially support verbal/episodic memory(Dinoff et al., 2018; Erickson et al., 2011). In contrast, improvements in visual or visuospatial memory rely more heavily on prefrontal-dependent pathways, such as enhanced cerebral perfusion, synaptic plasticity, and HPA-axis regulation (Stillman et al., 2016). The absence of a significant effect on visual memory in the present study may therefore reflect weaker engagement of these prefrontal-dependent mechanisms under the exercise conditions typically implemented in the included trials. Exercise may thus exert stronger effects on hippocampal-dependent verbal memory relative to visual memory, providing a biologically plausible explanation for the observed dissociation (Kandola et al., 2019).

### Characteristics of exercise

Due to the inability to categorize exercise frequency, this study focused exclusively on the effects of exercise type, session time, intensity, and total duration on verbal memory. Intensity (pre-specified as the primary moderator) showed a significant omnibus effect (*F* = 3.39, *p* = 0.04), with a small-to-moderate benefit for the low-to-moderate category (*g* = 0.43, 95% CI [0.16–0.71]); the other two intensity levels were not significant. Given the small magnitude and between-study uncertainty, this pattern should be interpreted cautiously. In contrast, the other two exercise intensity levels did not show improvements in verbal memory. Liu et al. (2024) also reached similar conclusions, finding that exercise intensity has a moderating effect on working memory in depressed patients, with low-intensity exercise being the most beneficial. However, other studies have reported different findings. Ren et al. (2023) and Ren et al. (2024) discovered that exercise intensity was not a moderating factor for improving executive function and cognitive abilities in depressed patients, suggesting that “moderate-to-vigorous” intensity produced the most favorable outcomes. These divergences likely reflect (i) differences in outcome domains—our analysis targets verbal memory specifically, whereas prior work examined executive or general cognition; (ii) different intensity categorizations (three levels here *vs.* two levels previously); (iii) analytic choices; and (iv) contextual features such as supervision, adherence, and tolerability. From a mechanistic standpoint, higher intensities may reduce affective valence and tolerability (Ekkekakis & Lind, 2006), potentially lowering adherence and diluting observable gains (Vandoni et al., 2016). Overall, the current evidence suggests a small, context-dependent advantage for verbal memory at low-to-moderate intensities, while alternative patterns reported elsewhere may stem from domain, categorization, and context differences rather than direct contradictions.

Exercise duration was not a moderating factor, and only “≤12 weeks” showed a small-to-moderate effect (*g* = 0.27). Ren et al. (2024) also posited that exercise for “≤12 weeks” can improve cognitive function in patients with depression. However, Liu et al. (2024) contended that exercise lasting 12–16 weeks is necessary to enhance working memory in depressed patients, while Ren et al. (2023) suggested that exercise for “≥13 weeks” is required to improve executive function. This discrepancy may be attributed to differences in outcome measures. Although our study indicated that exercise of “≤12 weeks” is effective, the studies we included had a minimum exercise duration of 3 weeks. Some research indicates that significant improvements in depressive symptoms often occur within the initial weeks of exercise participation (Zhao, 2014). Conversely, excessively prolonged exercise regimens may lead to boredom and frustration in patients with depression, resulting in increased dropout rates.

Session time was not a moderating factor, and only “≤60 minutes” demonstrated a small effect (*g* = 0.18). This finding is consistent with previous research. Ren et al. (2023) and Ren et al. (2024) indicated that “≤60 minutes” can enhance executive function and cognitive abilities in patients with depression. Prolonged exercise durations may lead to feelings of fatigue, dehydration, and disruptions in neuronal homeostasis, ultimately impairing cognitive function (Liu & Zeng, 2015).

Exercise type was not a moderating factor, with only “Mind–Body Exercise” demonstrating a small-to-moderate effect (*g* = 0.43). This result aligns closely with those of Liu et al. (2024) and Sun et al. (2018). Conversely, Ren et al. (2023) posited that only aerobic exercise can enhance executive function in patients with depression. We propose that “Mind–Mind-body exercise”, which includes practices such as yoga and tai chi, integrates physical postures, breath regulation, and relaxation techniques to achieve a dynamic balance between the body, mind, and external environment. This integration may be particularly effective in alleviating negative emotions (Yang et al., 2019). Moreover, these practices require attention and involve multiple cognitive processes, such as visual-spatial awareness and memory, to maintain stable body postures, thereby potentially enhancing perceptual functions and cognitive abilities, including memory (Zou et al., 2019).

From a clinical standpoint, the present findings suggest that low-to-moderate intensity exercise performed for ≤60 min per session over ≤12 weeks may offer small but potentially meaningful benefits for verbal memory in adults with depression. Mind–body modalities such as yoga or tai chi may be especially suitable for individuals with low motivation or limited tolerance for higher-intensity activity. These patterns provide preliminary guidance for clinicians designing exercise prescriptions tailored to patients’ cognitive needs and functional capacities.

### Study design and sample characteristics

Subgroup analysis was performed on the intervention content of the experimental group and the age of the patients with depression. The intervention content was not identified as a moderating factor, with only “exercise combined with other therapies” demonstrating a small-to-moderate effect (*g* = 0.31). Previous studies have reported similar findings. Solely engaging in exercise did not improve the overall cognitive status of patients with depression, nor did it enhance specific cognitive dimensions such as attention and memory (Sun et al., 2018). Additionally, research indicates that combining antidepressant medication with appropriately intense aerobic exercise may more effectively ameliorate cognitive symptoms in patients with depression (Greer et al., 2015; Olson et al., 2017).

Age was not a moderating factor, with only “young adults (18–44 years)” demonstrating a small-to-moderate effect (*g* = 0.22). However, this finding is not consistent with previous research. Ren et al. (2023), Ren et al. (2024) indicated that exercise improved executive function and cognitive function only in “middle adults (45–59 years)” (Ren et al., 2023; Ren et al., 2024). Additionally, other studies have shown that exercise can enhance executive function in older adults aged 60 and above with mild cognitive impairment (Brandt et al., 2009). This discrepancy may be related to the limited number of studies involving participants over 65 years of age in the current research, as only two studies were included. Therefore, future investigations should focus more on the memory of elderly patients with depression. Furthermore, this may also relate to differences in outcome measures, as prior studies have placed greater emphasis on executive function and overall cognitive performance.

### Strengths and limitations

This study has several advantages. It is the first to utilize a three-level meta-analysis to investigate the effects of exercise on verbal and visual memory in patients with depression, predicting a 95% CI, and it is the first to find no improvement in visual memory. Additionally, this research builds upon prior studies by expanding the literature search and including grey literature to mitigate publication bias, enhancing the reliability of the results. Furthermore, the study explores various factors affecting memory, providing evidence-based support for the development of exercise interventions targeting memory in depressed patients.

However, several limitations should be noted. Although the three-level approach addresses the methodological limitations of traditional meta-analyses that assume independence among effect sizes, the small number of included studies and uneven subgroup sizes limited its statistical advantages. All subgroup analyses should therefore be interpreted as exploratory. Additionally, restricting the search to Chinese- and English-language publications introduces potential language bias and may limit global generalizability.

Considerable heterogeneity also existed across studies in terms of exercise protocols (type, supervision, progression), participant characteristics (depression severity, medication use, comorbidities), and measurement tools. These factors likely contributed to residual between-study variability and may have obscured differential responses to exercise. Furthermore, despite inclusion of Chinese literature, non-Western samples remained under-represented, highlighting the need for broader geographical diversity in future work.

Finally, limitations in available data prevented stratification by depression severity or examination of additional effect modifiers such as baseline cognitive status. Although multiplicity was controlled using the Benjamini–Hochberg FDR, false-positive findings cannot be entirely ruled out. Future RCTs should implement standardized memory assessments, evaluate dose–response relationships, and incorporate mechanistic biomarkers (*e.g.*, BDNF levels, HPA-axis indicators) to clarify pathways through which exercise influences memory.

## Conclusion

Exercise may confer a small improvement in verbal memory among adults with depression, while no clear effect was observed for visual memory. Mind-body exercises of low-to-moderate intensity, with a duration of ≤12 weeks and sessions lasting ≤60 min, are most likely to benefit verbal memory in these patients. Future research is needed to explore further the effects of exercise on memory in patients with depression across varying severity levels and to clarify the dose–response relationship between exercise interventions and memory outcomes, aiming to develop optimal exercise protocols.

## Supplemental Information

10.7717/peerj.20750/supp-1 Supplemental Information 1 Supplemental materials Conceptual Model, Leave-one-out analysis and justification for conducting this systematic review and meta-analysis

10.7717/peerj.20750/supp-2 Supplemental Information 2 PRISMA checklist

10.7717/peerj.20750/supp-3 Supplemental Information 3 Data and R code

10.7717/peerj.20750/supp-4 Supplemental Information 4 Translation codebook

## Additional Information and Declarations

### Data Availability

The following information was supplied regarding data availability: The raw data is available in the Supplemental Files.
