# Supplementary material for: Effects of exercise interventions on memory in depression: a three-level meta-analysis
Source: PeerJ. 2026 Feb 5;14:e20750. doi: 10.7717/peerj.20750 (PMC12883154; doi:10.7717/peerj.20750)
Supplement: Supplemental Information 1 — Conceptual Model, Leave-one-out analysis and justification for conducting this systematic review and meta-analysis [file peerj-14-20750-s001.docx]

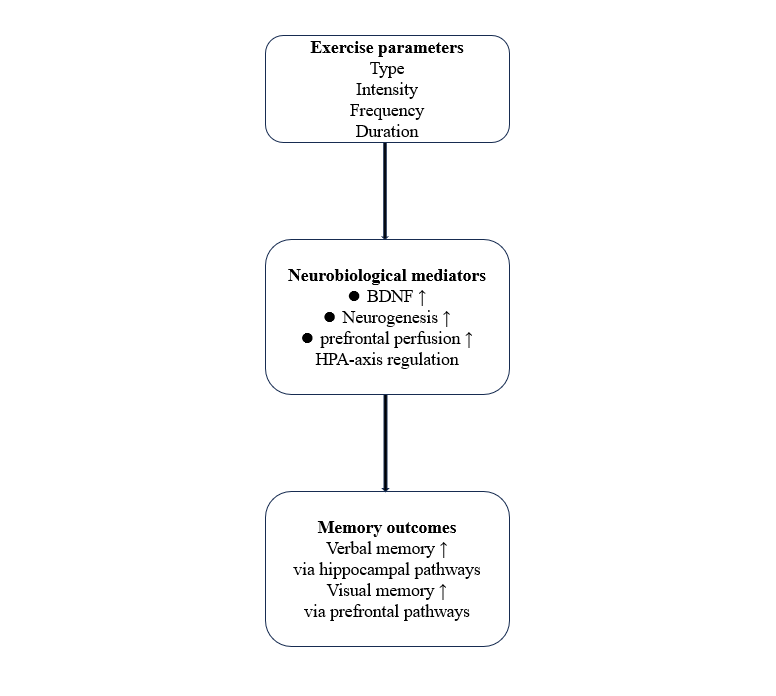


Figure 1. Conceptual Model

Table 1. Leave-one-out analysis for verbal memory

| name | estimate | se | tval | pval | ci.lb | ci.ub | Q | Qp | tau2 | I2 | H2 |
| --- | --- | --- | --- | --- | --- | --- | --- | --- | --- | --- | --- |
| Halappa2018 Yoga with medication-RAVLTTS | 0.17 | 0.07 | 2.35 | 0.02 | 0.02 | 0.31 | 13.10 | 1.00 | 0.00 | 0.00 | 1.00 |
| Halappa2018 Yoga-RAVLTTS | 0.17 | 0.07 | 2.37 | 0.02 | 0.03 | 0.31 | 13.14 | 1.00 | 0.00 | 0.00 | 1.00 |
| Halappa2018 Yoga with medication-RAVLTDLB | 0.17 | 0.07 | 2.33 | 0.02 | 0.02 | 0.31 | 12.85 | 1.00 | 0.00 | 0.00 | 1.00 |
| Halappa2018 Yoga-RAVLTDLB | 0.16 | 0.07 | 2.29 | 0.03 | 0.02 | 0.31 | 11.80 | 1.00 | 0.00 | 0.00 | 1.00 |
| Halappa2018 Yoga with medication-RAVLTIR | 0.17 | 0.07 | 2.33 | 0.02 | 0.02 | 0.31 | 12.85 | 1.00 | 0.00 | 0.00 | 1.00 |
| Halappa2018 Yoga-RAVLTIR | 0.17 | 0.07 | 2.43 | 0.02 | 0.03 | 0.32 | 13.57 | 1.00 | 0.00 | 0.00 | 1.00 |
| Halappa2018 Yoga with medication-RAVLTIRDR | 0.17 | 0.07 | 2.41 | 0.02 | 0.03 | 0.32 | 13.53 | 1.00 | 0.00 | 0.00 | 1.00 |
| Halappa2018 Yoga-RAVLTIRDR | 0.17 | 0.07 | 2.38 | 0.02 | 0.03 | 0.31 | 13.31 | 1.00 | 0.00 | 0.00 | 1.00 |
| Halappa2018 Yoga with medication-DSTF | 0.17 | 0.07 | 2.37 | 0.02 | 0.03 | 0.31 | 13.25 | 1.00 | 0.00 | 0.00 | 1.00 |
| Halappa2018 Yoga-DSTF | 0.17 | 0.07 | 2.36 | 0.02 | 0.03 | 0.31 | 13.11 | 1.00 | 0.00 | 0.00 | 1.00 |
| Halappa2018 Yoga with medication-DSTB | 0.17 | 0.07 | 2.45 | 0.02 | 0.03 | 0.32 | 13.59 | 1.00 | 0.00 | 0.00 | 1.00 |
| Halappa2018 Yoga-DSTB | 0.17 | 0.07 | 2.41 | 0.02 | 0.03 | 0.32 | 13.48 | 1.00 | 0.00 | 0.00 | 1.00 |
| Lavretsky2011 | 0.17 | 0.07 | 2.37 | 0.02 | 0.03 | 0.31 | 13.36 | 1.00 | 0.00 | 0.00 | 1.00 |
| Lavretsky2022 | 0.17 | 0.07 | 2.40 | 0.02 | 0.03 | 0.32 | 13.56 | 1.00 | 0.00 | 0.00 | 1.00 |
| Khatri2001 WMSLMIR | 0.18 | 0.07 | 2.48 | 0.02 | 0.03 | 0.32 | 13.52 | 1.00 | 0.00 | 0.00 | 1.00 |
| Khatri2001 WMSLMDR | 0.18 | 0.07 | 2.51 | 0.02 | 0.04 | 0.32 | 13.39 | 1.00 | 0.00 | 0.00 | 1.00 |
| Khatri2001 DSTF | 0.19 | 0.07 | 2.60 | 0.01 | 0.04 | 0.33 | 12.53 | 1.00 | 0.00 | 0.00 | 1.00 |
| Khatri2001 DSTB | 0.19 | 0.07 | 2.61 | 0.01 | 0.04 | 0.33 | 12.34 | 1.00 | 0.00 | 0.00 | 1.00 |
| Imboden2020 | 0.17 | 0.07 | 2.39 | 0.02 | 0.03 | 0.31 | 13.37 | 1.00 | 0.00 | 0.00 | 1.00 |
| Hoffman2008 WMSLM | 0.18 | 0.07 | 2.54 | 0.01 | 0.04 | 0.33 | 13.17 | 1.00 | 0.00 | 0.00 | 1.00 |
| Hoffman2008 WMSVPE | 0.17 | 0.07 | 2.39 | 0.02 | 0.03 | 0.32 | 13.54 | 1.00 | 0.00 | 0.00 | 1.00 |
| Hoffman2008 WMSVPH | 0.18 | 0.07 | 2.45 | 0.02 | 0.03 | 0.32 | 13.58 | 1.00 | 0.00 | 0.00 | 1.00 |
| Hoffman2008 DSTF | 0.18 | 0.07 | 2.49 | 0.02 | 0.03 | 0.32 | 13.50 | 1.00 | 0.00 | 0.00 | 1.00 |
| Hoffman2008 DSTB | 0.18 | 0.07 | 2.52 | 0.02 | 0.04 | 0.33 | 13.34 | 1.00 | 0.00 | 0.00 | 1.00 |
| Krogh2009 Strenght DS | 0.18 | 0.07 | 2.44 | 0.02 | 0.03 | 0.32 | 13.59 | 1.00 | 0.00 | 0.00 | 1.00 |
| Krogh2009 Aerobic DS | 0.18 | 0.07 | 2.52 | 0.02 | 0.04 | 0.32 | 13.32 | 1.00 | 0.00 | 0.00 | 1.00 |
| Krogh2009 Strenght BSRT | 0.18 | 0.07 | 2.51 | 0.02 | 0.04 | 0.32 | 13.37 | 1.00 | 0.00 | 0.00 | 1.00 |
| Krogh2009 BSRT | 0.18 | 0.07 | 2.55 | 0.01 | 0.04 | 0.33 | 13.10 | 1.00 | 0.00 | 0.00 | 1.00 |
| Krogh2012 BSRT | 0.18 | 0.07 | 2.52 | 0.02 | 0.04 | 0.33 | 13.31 | 1.00 | 0.00 | 0.00 | 1.00 |
| Krogh2012 DSTF | 0.18 | 0.07 | 2.52 | 0.02 | 0.04 | 0.33 | 13.32 | 1.00 | 0.00 | 0.00 | 1.00 |
| Krogh2012 DSTB | 0.18 | 0.07 | 2.54 | 0.01 | 0.04 | 0.33 | 13.17 | 1.00 | 0.00 | 0.00 | 1.00 |
| Krogh2014 BSRT | 0.18 | 0.07 | 2.50 | 0.02 | 0.03 | 0.32 | 13.45 | 1.00 | 0.00 | 0.00 | 1.00 |
| Oertel-Knochel2014 Verbal learning | 0.18 | 0.07 | 2.46 | 0.02 | 0.03 | 0.32 | 13.59 | 1.00 | 0.00 | 0.00 | 1.00 |
| Bushert2019 WMSRSTM | 0.17 | 0.07 | 2.34 | 0.02 | 0.02 | 0.31 | 12.65 | 1.00 | 0.00 | 0.00 | 1.00 |
| Bushert2019 VLMTLT | 0.18 | 0.07 | 2.52 | 0.02 | 0.04 | 0.32 | 13.21 | 1.00 | 0.00 | 0.00 | 1.00 |
| Bushert2019 VLMTRE | 0.18 | 0.07 | 2.47 | 0.02 | 0.03 | 0.32 | 13.57 | 1.00 | 0.00 | 0.00 | 1.00 |
| Bushert2019 DSTB | 0.17 | 0.07 | 2.43 | 0.02 | 0.03 | 0.32 | 13.57 | 1.00 | 0.00 | 0.00 | 1.00 |
| Zheng2019 Immediate recall | 0.17 | 0.07 | 2.38 | 0.02 | 0.03 | 0.31 | 13.40 | 1.00 | 0.00 | 0.00 | 1.00 |
| Zheng2019 Delay recall | 0.17 | 0.07 | 2.42 | 0.02 | 0.03 | 0.32 | 13.57 | 1.00 | 0.00 | 0.00 | 1.00 |
| Sharma2006 DSTF | 0.17 | 0.07 | 2.42 | 0.02 | 0.03 | 0.32 | 13.51 | 1.00 | 0.00 | 0.00 | 1.00 |
| Sharma2006 DSTB | 0.18 | 0.07 | 2.47 | 0.02 | 0.03 | 0.32 | 13.57 | 1.00 | 0.00 | 0.00 | 1.00 |
| Chen2021 DSTF 16week | 0.17 | 0.07 | 2.35 | 0.02 | 0.02 | 0.31 | 13.34 | 1.00 | 0.00 | 0.00 | 1.00 |
| Chen2021 DSTB 16week | 0.18 | 0.07 | 2.45 | 0.02 | 0.03 | 0.32 | 13.58 | 1.00 | 0.00 | 0.00 | 1.00 |
| Chen2021 DSTF 8week | 0.18 | 0.07 | 2.47 | 0.02 | 0.03 | 0.32 | 13.55 | 1.00 | 0.00 | 0.00 | 1.00 |
| Chen2021 DSTB 8week | 0.17 | 0.07 | 2.43 | 0.02 | 0.03 | 0.32 | 13.59 | 1.00 | 0.00 | 0.00 | 1.00 |
| Zhang2019 DSTF 4week | 0.18 | 0.07 | 2.47 | 0.02 | 0.03 | 0.32 | 13.55 | 1.00 | 0.00 | 0.00 | 1.00 |
| Zhang2019 DSTF 8week | 0.18 | 0.07 | 2.46 | 0.02 | 0.03 | 0.32 | 13.59 | 1.00 | 0.00 | 0.00 | 1.00 |
| Zhang2019 DSTB 4week | 0.17 | 0.07 | 2.43 | 0.02 | 0.03 | 0.32 | 13.58 | 1.00 | 0.00 | 0.00 | 1.00 |
| Zhang2019 DSTB 8week | 0.17 | 0.07 | 2.38 | 0.02 | 0.03 | 0.31 | 13.33 | 1.00 | 0.00 | 0.00 | 1.00 |

Table 2. Leave-one-out analysis for spatial memory

| name | estimate | se | tval | pval | ci.lb | ci.ub | Q | Qp | tau2 | I2 | H2 |
| --- | --- | --- | --- | --- | --- | --- | --- | --- | --- | --- | --- |
| Khatri2001 WMSVRIR | 0.24446417 | 0.13039202 | 1.87483997 | 0.08345615 | -0.0372307 | 0.526159 | 2.97814801 | 0.99801194 | 0 | 0 | 1 |
| Khatri2001 WMSVRDR | 0.24491141 | 0.13039399 | 1.87824163 | 0.08295891 | -0.0367877 | 0.52661049 | 2.99429797 | 0.99795479 | 0 | 0 | 1 |
| Hoffman2008 Ruff | 0.29231213 | 0.13182684 | 2.21739463 | 0.04503421 | 0.00751755 | 0.57710671 | 2.94428696 | 0.99812778 | 0 | 0 | 1 |
| Krogh2009 Strenght RCFT | 0.29817955 | 0.13062168 | 2.28277226 | 0.0399135 | 0.01598856 | 0.58037053 | 2.5694178 | 0.99909477 | 0 | 0 | 1 |
| Krogh2009 Aerobic RCFT | 0.27223806 | 0.13064119 | 2.0838608 | 0.05746996 | -0.0099951 | 0.55447119 | 3.34423456 | 0.99637968 | 0 | 0 | 1 |
| Krogh2012 RCFT | 0.24626116 | 0.13127075 | 1.87597899 | 0.08328935 | -0.037332 | 0.52985437 | 3.0934391 | 0.99757599 | 0 | 0 | 1 |
| Krogh2014 RCFT | 0.25919637 | 0.13028944 | 1.98938894 | 0.06812155 | -0.0222769 | 0.5406696 | 3.32966772 | 0.99645934 | 0 | 0 | 1 |
| Oertel-Knochel2014 Visual learning | 0.26342849 | 0.12753898 | 2.06547441 | 0.05941371 | -0.0121027 | 0.5389597 | 3.35492312 | 0.99632037 | 0 | 0 | 1 |
| Zheng2019 DS | 0.26868991 | 0.12969104 | 2.07176929 | 0.05874147 | -0.0114905 | 0.54887036 | 3.36646615 | 0.99625551 | 0 | 0 | 1 |
| Chen2021 8week VRS | 0.27430626 | 0.13160641 | 2.08429249 | 0.05742504 | -0.0100121 | 0.55862463 | 3.32990597 | 0.99645805 | 0 | 0 | 1 |
| Chen2021 16week VRS | 0.28602063 | 0.13161819 | 2.17310874 | 0.04884796 | 0.00167683 | 0.57036444 | 3.11724992 | 0.99747755 | 0 | 0 | 1 |
| Zhang2019 4week VRS | 0.27218792 | 0.12901086 | 2.10980634 | 0.05482741 | -0.0065231 | 0.55089893 | 3.33103232 | 0.99645193 | 0 | 0 | 1 |
| Zhang2019 8week VRS | 0.27548978 | 0.12901418 | 2.13534493 | 0.05233721 | -0.0032284 | 0.55420798 | 3.27236651 | 0.99675999 | 0 | 0 | 1 |
| Zhang2022 4week TMS | 0.25424426 | 0.12884121 | 1.97331479 | 0.07010537 | -0.0241002 | 0.53258877 | 3.1924084 | 0.99714682 | 0 | 0 | 1 |
| Zhang2022 8week TMS | 0.24383555 | 0.12879132 | 1.89326067 | 0.08079543 | -0.0344012 | 0.52207229 | 2.73692978 | 0.99872869 | 0 | 0 | 1 |


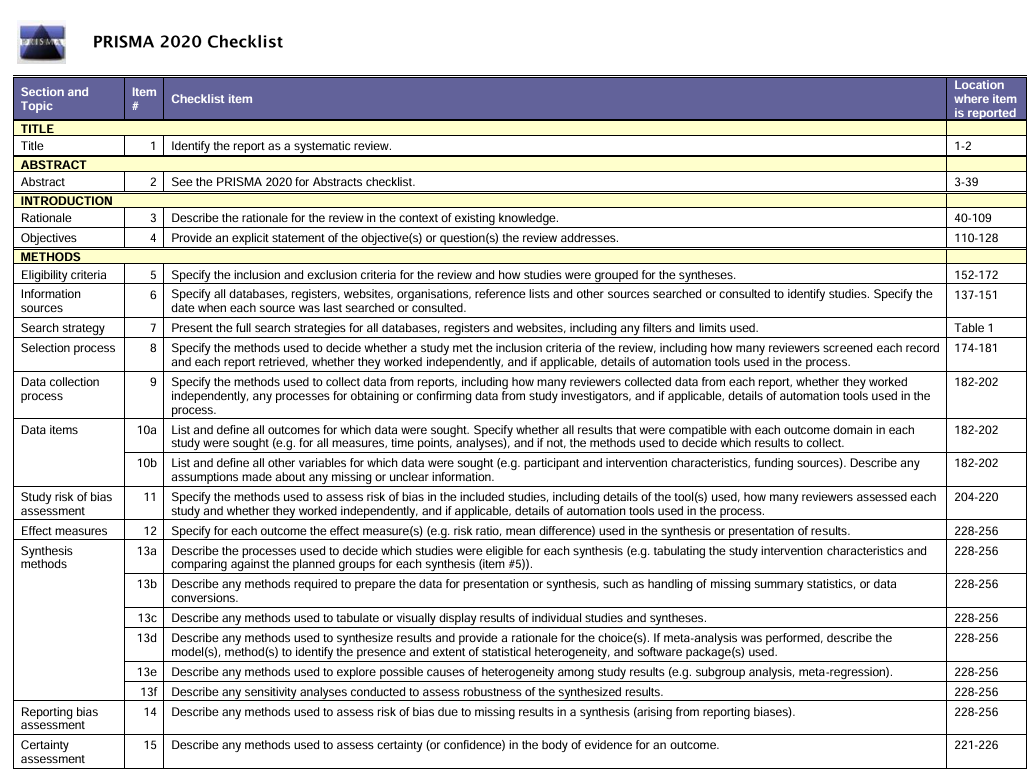


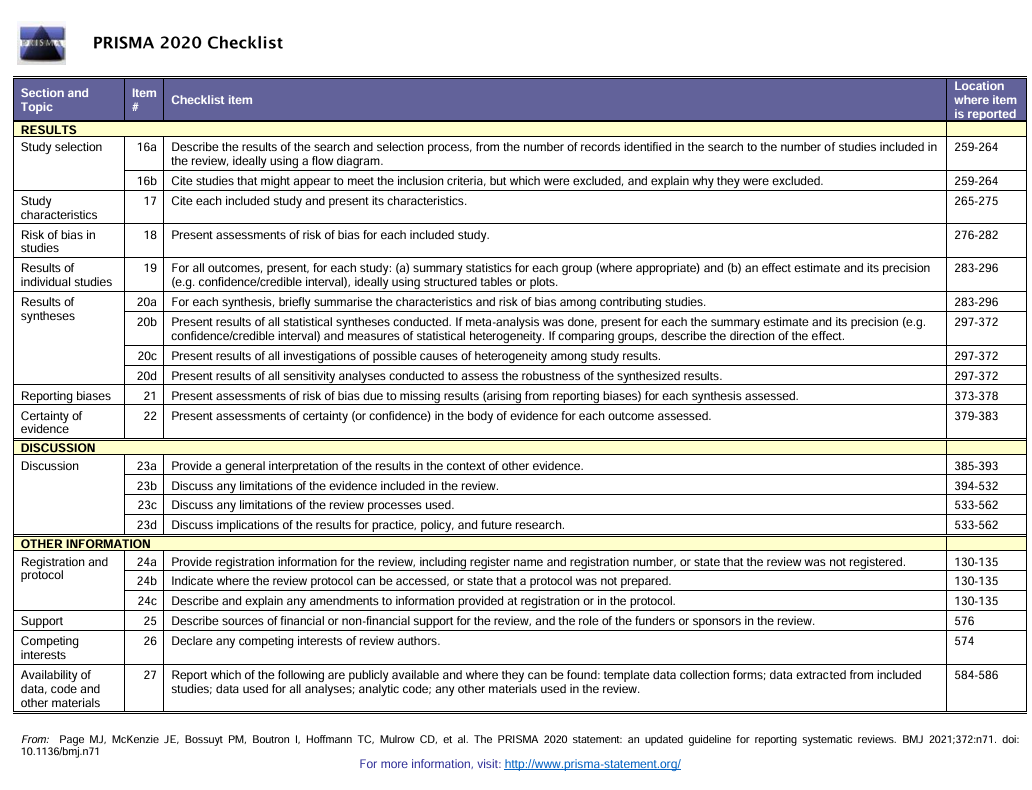


Justification for Conducting This Systematic Review and Meta-analysis

This systematic review and meta-analysis was conducted in accordance with the PeerJ policy requiring authors to justify the need for a new quantitative synthesis. The rationale for undertaking this review is as follows:

Lack of recent and comprehensive evidence synthesis

Although several previous reviews have examined exercise-related interventions in patients with depression, none has specifically focused on the effects of exercise on memory outcomes in clinically diagnosed depressive patients. Existing reviews either combine multiple cognitive domains, pool heterogeneous populations, or include non-clinical samples, thereby limiting the ability to draw precise conclusions regarding memory-specific effects.

Heterogeneity in exercise modalities and intervention characteristics

Previous reviews have not systematically compared different exercise types, intensities, frequencies, and durations in relation to memory outcomes. Our review aims to refine the understanding of intervention characteristics by examining whether specific exercise components show differential effects on memory in depressed populations.

Need for updated evidence based solely on randomized controlled trials

Earlier reviews often included quasi-experimental or observational studies, increasing risk of bias. The current review restricts inclusion to randomized controlled trials (RCTs), providing stronger causal inference.

Advances in methodological rigor

Prior studies commonly used traditional two-level meta-analytic techniques. Our work applies updated methods, incorporates RoB 2.0 for risk-of-bias assessment, and provides GRADE certainty of evidence ratings, thereby improving transparency and methodological strength.

Clinical significance and knowledge gap

Memory impairment is one of the most debilitating cognitive symptoms associated with depression, yet no prior synthesis has isolated memory performance as the primary outcome. This review fills an important gap and provides clinicians with clearer evidence on whether exercise should be considered as an adjunct treatment for memory deficits in depression.

Based on these reasons, a new systematic review and meta-analysis is warranted and contributes novel evidence to the field.
