# Supplementary material for: Effects of exercise interventions on memory in depression: a three-level meta-analysis
Source: PeerJ. 2026 Feb 5;14:e20750. doi: 10.7717/peerj.20750 (PMC12883154; doi:10.7717/peerj.20750)
Supplement: Supplemental Information 4 [file peerj-14-20750-s004.docx]

Translations for non-English text used in data_and_R.rar

1. #设置工作路径

**Translation: # Set working directory**

1. 加载Matrix

**Translation: # Load Matrix**

1. 加载metafor

**Translation: #Load metafor**

1. 导入数据

**Translation: Import data**

1. 查看数据

**Translation: View data**

1. 计算整体效果（影响力识别）（Three level）

**Translation: Calculate overall effect (influence identification) (Three-level model)**

1. two level model

**Translation: Two-level model**

1. 写入 Excel

**Translation: Export to Excel**

1. 异质性分析（Level 2）

**Translation: Heterogeneity analysis (Level 2)**

1. 异质性分析（Level 3）

**Translation: Heterogeneity analysis (Level 3)**

1. 调节因素

**Translation: Moderator analysis**

1. young, middle, old

**Translation: young adults (18-44 years), middle-aged adults (45-64 years), older adults (≥ 65 years)**

1. duration, shcyc, longcyc

**Translation: exercise duration, shcyc(≤12 weeks) and longcyc(>12 weeks)**

1. type, Aero, Resis, Mindbody

**Translation: exercise type, Aerobic exercise, strength exercise, and mind-body exercise.**

1. time, Shorttime, Longtime

**Translation: exercise time, Shorttime (≤60 minutes), Longtime(>60 minutes).**

1. Intensity, Lowmid, mid, Vig

**Translation:exercise intensity, low-to-moderate, moderate, moderate-to-vigorous.**

1. content, only, combine

**Translation: the intervention content of the experimental group, exercise alone, exercise combined with other therapies**

1. 发表偏倚

**Translation: Publication bias assessment**

1. 轮廓增强漏斗图

**Translation: contour-enhanced funnel plot**
